# Report of Committee on Practical Medicine

**Published:** 1864-06

**Authors:** N. S. Davis

**Affiliations:** Prof. of Practical and Clinical Medicine


### CHICAGO MEDICAL EXAMINER.

N. S. DAVIS, M.D., Editor.

VOL. V. JUNE, 1864. NO. 6.

### Original Contributions.

ARTICLE XXIII.

### REPORT OF COMMITTEE ON PRACTICAL MEDICINE.

By N. S. DAVIS, M.D., Prof. of Practical and Clinical Medicine.

Presented to the Illinois State Medical Society, May, 1864.

By the constitution, it is made the duty of the Committee on Practical Medicine to report at each Annual Meeting of the Society on such epidemic diseases as may have prevailed, and on such improvements in the department of Practical Medicine as may have been made in this State during the preceding year. In regard to such epidemics as have prevailed within the limits of this State during the past year, your Committee have not as full information as is desirable. Those chiefly worthy of attention are erysipelas and cerebro-spinal meningitis or Spotted Fever. In the city of Chicago, sporadic cases of erysipelas occurred rather more frequently than usual during all the summer of 1863. During the month of September, the cases became so much more numerous as to attract the attention of the profession; and from that time to the first of March the prevalence of the disease was such as to fully merit the title of epidemic. During the six months ending March 1st, 1864, twenty-one cases of this disease were admitted into the medical wards of the Mercy Hospital. In nearly all of these cases the disease commenced with rigors and all the ordinary symptoms of continued fever, with some soreness of the fauces. In from one to three days, the erysipelatous inflammation made its appearance on the face—generally near the alæ nasi—though in a few cases it commenced at the lobe of the ear. It usually spread rapidly over the entire face and scalp, and, in a few cases, down the neck to the upper part of the trunk. In most of the cases there was much tumefaction of the face, extensive vesications, and, in three cases, suppuration in the cellular tissue of the eyelids. Nearly all of these patients come from the poorer classes of society in the city, and had been laboring under the disease from two to five days before their admission. The grade of fever accompanying these cases was decidedly typhoid; the pulse being frequent, soft, and quick; the skin dry; the inflamed surface and fauces dark red; mind dull and sometimes wandering; the tongue covered with a redish-brown coat and inclined to be dry; urine dark and scanty; and the bowels easily moved.

In a few cases the stomach was irritable, but not generally; and in four of the most severe cases there were frequent intestinal discharges of a thin redish-brown character.

*Treatment.*—The four cases last mentioned were treated exclusively with sulphite of lime in doses of from half a drachm to a drachm, repeated every two hours during the first day, and subsequently every three or four hours, until convalescence was established. To control the intestinal irritation, a teaspoonful of the following emulsion was given between each of the doses of sulphite of lime, viz.:—

℞. Ol. Terebinth . . ʒjj

Tinct. Opii . . . ʒjj

Pulv. G. Acaciæ }

Sacchar. Alba } āā ʒjjj—Rub together.

And add—Aqua Menthæ . . ℥jj—Mix.

All the other cases were treated almost exclusively with the tinct. ferri chloridi, in doses of from 20 to 30 drops, repeated every two hours until the external inflammation ceased to spread, and then every three or four hours, until convalesence was established. In three or four cases in which there was troublesome gastric irritation at the time of admission, the treatment was commenced with four or five alterative doses of calomel with morphine.

In a few cases, moderate doses of quinine and opium were given to procure more rest at night. In all the cases, nourishment, in the form of animal broths or milk, was regularly administered, but no diffusable stimulants except tea or coffee. The time required to establish convalescence, varied from four to twelve days.

In fourteen of the twenty-one cases, the recovery after convalescence was rapid and attended by no unpleasant sequelæ. In the remaining seven it was more slow and tedious. Two had small cellular abscesses in the eyelids; one, a very protracted ottorrhœa, with partial deafness and giddiness; and one had three distinct relapses or renewals of the erysipelatous inflammation, each time extending over the face, ears, and mastoid spaces. No deaths occurred from this disease in the Hospital. During the time the foregoing cases were under treatment in the Hospital, namely, the six months ending with the first of March, 1864, there came under my care, in private practice, in this city, forty-five additional cases of erysipelas. These were treated in the same manner as those admitted into the Hospital, and all except one recovered. The case that terminated fatally, was a female child only *four weeks* old. The erysipelatous inflammation attacked the vulva and nates, from which it extended gradually over the entire cutaneous surface, from the toes to the top of the head. It was accompanied by both vomiting and diarrhœa. It terminated fatally in eight or nine days after the attack. The whole number of cases that occurred in the city, during this epidemic, we have no means of ascertaining.

*Causes.*—No department of medical investigations is environed with more difficulties than that of etiology. Some have attributed the unusual prevalence of erysipelas in this city, during the past autumn and winter, to the impure and offensive condition of the Chicago river. It is certainly a fact, that, at the time the erysipelas became sufficiently prevalent to attract attention, the Chicago river was in an extremely offensive state. The pork and beef-packing business had commenced, and sufficient blood and offal had been precipitated into the river, in connexion with the ordinary sewerage matter, to render the whole water in the channel a dark redish-brown color, and to cause the emission of a putrefactive odor, perceptibly tainting the atmosphere to the extent of half a mile from the stream. From a careful inquiry concerning the particular parts of the city in which the cases were located, Prof. E. ANDREWS came to the conclusion that much the larger number occurred in streets approximating the river; while the few cases, occurring in parts of the city remote from the river, were mostly in persons whose places of business were on or near that stream. These facts point directly to the conclusion that the poisonous and offensive effluvia from the river constituted the efficient cause of the erysipelas. On the other hand, a more extended investigation shows that sporadic cases of erysipelas had been occurring in the city during all the last half of summer, and were becoming more frequent before the meat-packing season had commenced. We are also informed that the same disease has prevailed more than usual in several places in the interior of the State. Hence we were led to infer that, while the bad condition of the Chicago river and of the water supplied to families throughout the city, may have increased the prevalence and severity of the disease, they were not the primary causes of it.

From all the facts we have been able to gather, we are much disposed to think that the prevalence of erysipelas has been only one of the manifestations of a general or wide-spread epidemic influence, disposing to diseases of a decidedly *Typhus* type.

This view is confirmed by the fact, that, coincidently with the prevalence of erysipelas in this city, the Typhoid, Typhus, and Puerpural fevers, have also been unusually prevalent. Thus, during the six months ending March 1st, 1864, there were admitted, into the medical wards of the Mercy Hospital, *fifty* well-marked cases of Typhoid fever. Of these, six died and forty-four recovered. Of the six fatal cases, one was moribund at the time of admission, and died in twenty-four hours; there were admitted in the advanced stage of the disease, complicated with extensive pulmonary infiltration, and died during the first week after admission. The remaining two were under treatment, one thirty-eight and the other fifty-six days. Both died from local complications.

During the same period of time, there came under my care in private practice sixty-three cases of the same disease, of which two terminated fatally.

These figures certainly indicate a numerical prevalence of Typhoid fever in this city, considerably above the average for a series of years. Both from my own observation and from information furnished by other practitioners in the city, I am satisfied that true puerpural fever has also been much more prevalent during all the past winter than usual; and many of the cases have terminated fatally. Only five well-marked cases of this disease have occurred in my own practice, of which one died on the third day after the attack, and the remaining four recovered. About the same time that erysipelas was most prevalent, and when the water in the river and that furnished to the city through the hydraulic works, was extremely impure and offensive, namely, during the last half of October and the first of November, there occurred quite a number of cases of sickness of an unusual character, and a large proportion of which terminated speedily fatal.

Nearly all of these cases commenced with a harassing cough, moderate soreness in the air passages from the larynx to the bronchial ramifications, and a great sense of weakness or weariness. The pulse, during this stage, was not much disturbed, neither was the skin hot or dry; and the patients were able to be up and dressed. After these symptoms had continued from three to five or six days, there supervened suddenly a great sense of oppression across the chest, with great dyspnœa; a rapid pulse; cool extremities, and a rapidly accumulating tenacious mucus in the air passages. In six or eight of these cases the dyspnœa and apparent exhaustion increased so rapidly as to induce death by apnœa in from eighteen to twenty-four hours. Several of the cases occurred in good families, and in an advanced stage of pregnancy. These circumstances, with the unusual character and rapid progress of the attacks, caused them to attract much attention. What was the nature of these cases? Was it erysipelatous inflammation attacking primarily the mucous membrane of the bronchial tubes, and subsequently extending by continuity to the air-cells, inducing universal capillary congestion of the lungs and consequent suffocation?

I think so, although no opportunities could be obtained for making *post mortem* examinations.

Cerebro-Spinal Meningitis—Spotted Fever.—During the past year, and particularly during the last six months, a disease of rapid progress, and attended by a high ratio of mortality, has made its appearance in several localities in this State. There has been much diversity of opinion in regard to its nature, some regarding it as a specific local inflammation of the meninges of the base of the brain and of the medulla oblongata; some as a malignant form of scarlatina; while still others regard it as identical with the Spotted Fever or Sinking Typhus that prevailed in New-England extensively half a century since. It seems to have prevailed most in rural districts, and chiefly, thus far, in the central and southern parts of this State. Its attacks are not restricted to any class, sex, or age, although it seems to occur most frequently among the young. From the descriptions given by different practitioners whose opportunities for observation have been good, the disease varies in different localities, and in different cases in the same locality.

The following letter from my colleague, Prof. J. S. Jewell, will give an account of the disease as it prevailed in one of the most southern localities in the State. This letter is as follows:

Chicago, *May 2nd*, 1864.

Prof. N. S. Davis:

Dear Sir:—I give you, cheerfully, such information as I may have, in relation to the prevalence of epidemics, within the limits of this State.

During the past year, I have partially witnessed, and from others have learned, of two epidemics, one of "cerebro-spinal meningitis" or "spotted fever," as it is called; the other succeeding it, of erysipelas. The former occurred during the latter part of the winter of 1862 and '3, and in the spring which succeeded it,—since which time it has almost if not quite disappeared, with the exception that it reappeared last winter in a few localities, thus evincing a disposition to prevail during the winter, and to subside on the approach of warm weather; the *latter* during the summer of the year 1863, and especially during the autumn and winter succeeding,—the cases of the disease (*erysipelas*) multiplying as the season advanced, until by or before midwinter it raged in some localities with epidemic violence. In the latter part of the winter, the disease declined until at the present time it cannot be said to exist as an epidemic.

The district of country to which I refer, is in the southern part of the State, about 60 or 70 miles from Cairo, to the N.E. in the county of Williamson, and others adjoining, in a part of the country as a general thing level and somewhat low, though occasionally broken—furrowed by sluggish streams, which are frequently bordered by extensive alluvial bottoms, with marshes interspersed here and there, and as a general thing heavily timbered, except when removed for the purposes of agriculture or interrupted by occasional oak openings, or barrens, and small prairies.

Except during the wet season, the streams seldom run. The water employed for domestic purposes, is mostly obtained from cisterns, which all the rains of the year contribute to supply; or from wells, the water from which is in almost all cases impregnated with salts, obtained in the clays and soils through which it passes, known amongst the people as "hard water."

The district to which reference is had, presents, in a geological point of view, the sandstone formations, immediately overlaid by the coal measures and carboniferous shales, which in many cases lie so superficial as to be exposed—occasional masses of limestone—hard retentive clays—drift and alluvium.

It may not be improper to state that between the years of

1852 and 1855 an epidemic of erysipelatous ophthalmia occurred throughout that section of country, but few persons escaping an attack, and in many cases attended by the most deplorable results. In conjunction with this, miasmatic fevers prevailed to an extent never known there before,—this latter form of trouble reaching its highest degree of prevalence in 1855; and from this period to 1857, the autumnal fevers were almost the sole representations of this class of disease. From the period last mentioned, a transition from the periodical to the continued type of fevers occurred, until, in the course of two or three years, fevers of a continued type almost entirely supplanted the autumnal fevers. This state of things remained until, within the past year, a *return* is indicated to what may in general terms be called a more sthenic type, with an increased number of cases of miasmatic disorder, with the notable exceptions I have referred to in speaking of the epidemics, and a general tendency, exhibited in many cases and in different kinds of disease, in strict conformity with the plainer manifestations of epidemic influence.

Many of the cases of "spotted fever" which *first occurred*, were variously supposed to be inflammation of the brain, "sun-stroke," &c.; and it was not until some time had elapsed that its true character was apprehended. The disease naturally excited the utmost interest amongst both physicians and people. Most of the cases which *first* occurred, died—many of them too speedily to admit of any remedial action. It was not till some time after the advent of this disease that I had an opportunity of witnessing it. I have seen some eight cases in all, most of them, at some period in their progress, under my care. They were all violent cases, and, with two exceptions, all died, one or two without *any* medication.

I cannot, within the limits of this letter, detail cases, but can only give briefly the results of my observations. Since I commenced writing this, I have received, according to request, a communication from Drs. Lodge and Samuels, of Williamson County, who have practised in the midst of the epidemics, and who have kindly furnished me the results of their observations and experience.

All ages and both sexes fell victims to the disease. Children, however, seemed most liable to it. I did not observe that any marked premonitory symptoms served to show its approach. Drs. Lodge and Samuels noticed, however, pain along the course of the facial nerve and in the back of the head, with a slight redness of the eyes and intolerance to light as the earliest symptoms;—the person, as a general thing, feeling usually well, until at or about the period of attack, which, so far as I have seen, is ushered in by a chill—the duration of which varied from 30 minutes to 3 hours. In all the cases I observed, reaction followed, varying much in intensity, and more characterised by *excitement* than *strength.* Drs. Lodge and Samuels, however, saw cases where death occurred without reaction, the chill presenting to them the characteristics the *same* as in "pernicious fever." Sometimes the pulse was full, and but little accelerated. In the majority of cases, however, it was accelerated and diminished in strength—sometimes in volume.

There was nothing remarkable in the appearance either of the countenance or general surface—in most cases at *first*, and in some at no time. In *some*—perhaps *most* cases—at some period in the course of the disease, spots could be observed, neither regular as to the time nor place of their occurrence, and generally on those parts most remote from the central organs of circulation. However, they appeared sometimes on the trunk; and, if so, most frequently about the neck, breast, and shoulders. They were not permanent, and seemed to indicate *deficient capillary* circulation. With the exceptions mentioned, the circulatory system presented nothing remarkable; and in one case I observed, till death took place, the heart acted well and did not *cease* to act, until one or two minutes after respiration had ceased. The expression of countenance, if not that of distress, was dull and expressionless, or disfigured by convulsive action of the muscles of face. After the chill, the heat of surface was almost always above what is natural (till death in some cases); in others the heat of surface passed away, and generally with profuse perspiration.

As to the muscular system, there was generally restlessness from the beginning, followed by various irregular movements, and which, as the disease advanced with indications of coma, were increased to a greater or less degree in violence, especially in the case of children, where severe convulsions frequently occurred,—the attack varying in duration and frequency.

There were also permanent spasms of the voluntary muscles in all cases I saw, which affected *first* the extensor muscles of the back, subsequently the flexors of the limbs. The head, in most all cases, was thrown back, and *violently* so, after the full advent of the disease, and in one case remaining *so* till the end of the third week or longer. Of the muscles of the eyeball, the superior and internal recti-muscles were almost invariably contracted. According to Drs. Lodge and Samuels, the tongue was kept in constant motion, as if to remove something from the mouth. Of the muscles of respiration, the diaphragm was most frequently affected, the respiratory movements being thoracic. I had no particular evidence of trouble in the case of those muscles, supposed to be under the control of the organic nervous system. There was also, as I omitted to state, spasm of the muscles of mastication, and of the muscles of deglutition, in some cases, so that swallowing was difficult or impossible. Both sides of the body seemed in such cases equally affected. Tongue coated—whitish coat, but moist; bowels either constipated, or in good state; in other respects, nothing remarkable concerning digestive system. The secretions did not vary, in a general way, in any remarkable respect from the same in health. I regret to say I did not examine the urine, from want of proper facilities. Drs. Lodge and Samuels, however, observed it as scanty, at the outset, in many cases, and high-colored, and subsequently more copious, with an ammoniacal odor, and sometimes loaded with albumen.

As to the symptoms referable to the nervous system, they were by far the most important and significant.

There was in all cases pain in the head—generally in the back part—and extending down the neck and back, and occasionally excruciating in its character. Restlessness and delirium, or a disposition to it, which in most cases rapidly increased, were the most prominent symptoms, at the beginning of the attack—the restlessness increasing until the occurrence of convulsive movements of various degrees of violence. The delirium in most cases increased rapidly, and was succeeded by coma. The delirium was in some cases furious—dilated pupils —(though Drs. Lodge and Samuels sometimes saw them contracted)—heavy stertorous breathing, or, in some cases, short and hurried, and sibilant or *hissing* respiration, much increased by a rapid accumulation in the throat of mucus. Before loss of consciousness, there was frequently double vision, ringing and roaring sounds in the ears, with impairment, in various degrees, of the special senses. The order in which these latter symptoms sometimes occur, may be illustrated by reference to a case.

When I first saw the patient, he had been sick but two or three hours, and was, at the time referred to, partially conscious. Would respond, but not readily, to a question asked him; special and general sensation seemed not much impaired; in a few minutes he could not hear, as was evinced by calling him loudly. The pupils of the eyes were now much dilated, and did not respond to a strong light; nasal passages insensible to carb. of ammonia; and lips and tongue, to the tr. capsicum. General sensation seemed, if not gone, much impaired; and spasmodic muscular movements—ceased first in the upper, *last* in the lower, extremities—and in both sides simultaneously. Diaphragm apparently ceased to act. A few attempts at purely thoracic respiration was made, and the patient ceased to breathe. The heart, however, continued to act well to this period, and did not cease to act until nearly two minutes after respiration ceased. This, however, was not always, even in rapidly fatal cases, the manner of procedure. In nearly all cases, the *motor* function was *first* involved; the sensory *last*, and in various ways and degrees. In one case I observed increased sensitiveness of the surface, from the commencement of the attack until the end. The case recovered. In other cases, for a time, apparently, Drs. Lodge and Samuels observed the same in *most* of their cases.

The manner in which the symptoms referable to the nervous system occurred, left the impression on my mind that on the great ganglia at the base of the brain, but especially the medulla oblongata and the adjacent membranes, was expended the chief influence of the morbific agent, whatsoever it may be; and in most cases from *this*, as a centre, the unhealthy action invaded the spinal cord, on the one hand, and the brain proper on the other.

The course of the disease was most always rapid; though it might, in the more favorable cases, continue for weeks to terminate either fatally, in the end, as it frequently did, or in a slow recovery. Drs. Lodge and Samuels saw cases in which the disease, or its legitimate effects, remained for months before death or recovery. The prognosis, as I witnessed, was in almost all cases unfavorable, even after recovery seemed in view; but the physicians above-named were disposed to take a more favorable view from their experience. They distinguish two forms of the disease—one mild, without severe symptoms—referable to the nervous system, but in other respects resembling the more *severe* form of the disease. This latter form with them, as with others, has proved almost unmanageable, *unless seen early*, when there seems to be a much better chance for benefit. In the worst cases, from a state of apparent health the patient would experience the attack, and sometimes, in the space of two or three hours, would be dead. The disease appeared to be complicated with malarious influence, and most of the cases of any duration exhibited a decided periodical tendency. This seems to have been the case in an especial manner in the practice of the above-named physicians, and in the milder cases particularly. The epidemic, as they observed it, appeared in its greatest virulence in one of the most malarious districts in the range of their practice. The two epidemics (spotted fever and erysipelas) were commingled in the practice of these gentlemen occurring at the same time and in the same place, inducing in them the opinion that the two forms of disease were connected by some link common to both.

No opportunity, so far as I am aware, has been offered any one there to make an examination of the dead body. I had none. I examined slightly the blood of *one* patient, in particular. The most remarkable condition was the firmness of the coagulum and thickness of the fibrinous or *buffy coat* which, in a clot of two inches in thickness, occupied ¼ or ⅓ the entire thickness of the same.

The treatment found most beneficial consisted in outward applications of a highly stimulating character throughout the entire length of the spine, such as a saturated solution of camphor in oil of turpentine, blisters to the back of the neck and head, warm applications to the extremities, attention to the state of the bowels, alterative doses of mercury, in conjunction with camphor, valerian, ipecacuanha, nitrate potash, &c., and in a few cases quinia—particularly in those cases exhibiting a tendency to periodicity and at an early period in the disease. The quinia did not seem to answer in all cases, and in none so well in the latter stages of the disease as in the earlier.

In *all* the cases, whether mild or severe, Drs. Lodge and Samuels had occasion to treat, the *quinia* in *large* doses, in combination with full doses of *morphia*, seemed to be the most successful mode of treatment. After they had tried a long list of remedies—most all without any apparent benefit—the quinia and morphine are selected as the most efficient agents in its treatment. In some cases, however, it seemed of no avail. Brandy, wine, spts. nit. dulc., &c., were well borne, and in a general way all such measures as had a tendency to support and stimulate the nervous centres, in conjunction with *severe* revulsive measures, such seemed to be the most successful course for the onset of the disease; but, in cases of any duration, such treatment was not the most beneficial, after a few days, requiring a course more distinctly alterative and less stimulating, as some of the cases seemed to become acute, somewhat in character, if they passed on for several days or weeks, calling for a course of treatment much the same as that indicated in the latter stages of an attack of inflammation of the brain, with this difference, that the use of stimulants could not be dispensed with, though restricted in their use. Iodide potassium and stillingia, iodide of iron, mercurials, and conium, belladonna, hyoscyamus, &c., were all employed, either by Drs. Lodge and Samuels, or by myself—sometimes without benefit. Counter-irritants were employed throughout the disease, and were in a high degree beneficial, when *freely* employed. Sedatives, and especially those supposed to exercise such an influence over the brain and spinal cord, did not do well, nor, as a general thing, *any* sedative measures. Blood-letting I tried, but did not find any benefit, but rather harm; nor did cold to the head answer well in my experience, though it appears Drs. Lodge and Samuels regarded it more favorably.

Sulphite of lime was tried in one or two cases, and the muriated tincture of iron, but without any appreciable effect. They were administered, however, in conjunction with other remedies. This I did under the apprehension that the disease might be erysipelatous in character. Strychnia was administered in the latter stages of some cases, with the view of relieving the local or the general paralysis which constituted one of the sequelæ of the disease, and with ordinary benefit.

The epidemic of erysipelas occurred within the year 1863, and especially in the latter part of the year, and was somewhat peculiar in character. Its favorite seat seemed to be the mucous membrane of the throat, from this point extending to the nostrils on the one hand, and from thence to the face and head externally, and occasionally to the larynx. Such cases very frequently died—some of them, the diseased action appearing to pass through the cribriform plate of the ethmoid, and in this way extending to the membranes of the brain. Though this was the most usual seat of the disease, it attacked indifferently any part of the body and all ages.

In localities where the epidemic influence was most conspicuous, cases of puerpural fever occurred after nearly every case of parturition, and such cases almost invariably died. Most of the cases of erysipelas were easily managed, and in all respects the treatment was that which experience has declared best as a general thing in such cases.

I have not been able to obtain as full information concerning these matters as I could desire, nor could I give it within the limits of this letter.

I am, Dear Sir,

Yours truly,

JAS. STEWART JEWELL.

DR. HINCKLEY, of Leland, in the central part of the state, attributes to the disease, as it appeared in that locality during the past winter, substantially the same symptoms. All the accounts we have seen concerning it, agree that it is characterized by no special premonitory symptoms, but is generally ushered in by a chill; pain in the head, neck, and extremities; rapid impairment of the special senses, with loss of strength; frequent pulse; hot skin; rigidity of the muscles of the neck, jaws, and sometimes of the extremities; red or purple spots on the surface; frequently convulsive movements followed speedily, by coma and death.

The duration of the disease is generally short, many patients dying within twenty-four hours, while others linger many weeks. In the most malignant cases, the chill and pain in the back of the head is followed almost immediately by loss of vision; rapidly-increasing stupor; rigidity of the muscles of the neck; convulsive movements or paralysis of the extremities; irregular respiration; very frequent or variable pulse; general suppression of secretion; capillary congestion or petechial spots in the skin; and, in from six to twenty-four hours, death. In attacks of less severity, the chill is followed by febrile reaction of greater or less intensity; rigidity of muscles, especially of the neck, back, and jaws; extreme restlessness; mental dulness; red or purple spots on the surface; diminished, but not suppressed secretions; impaired muscular action, which, in the progress of the disease, sometimes amounts to paralysis, especially of the sphincters, thereby allowing dribbling of urine and involuntary focal evacuation.

In the advanced stages, the pain is sometimes transferred suddenly from the head to the extremities; and the final convalescence of the patient is slow and tedious.

*Diagnosis.*—Most of those who have written about this disease, claim that the diagnosis is easy. From typhus, or any form of continued fever, it differs in the absence of premonitory symptoms; in the suddenness of the attack; in the well-marked chill; in the muscular rigidity; in the greater intensity and more circumscribed character of the pain; in the more immediate impairment of secretion and organic changes; in the earlier appearance of red or petechial spots; in its disposition to attack the young; and in the absence of the odor usually belonging to typhus. Yet Dr. North, who wrote a very interesting paper on "Spotted Fever" in 1812, and most of the earlier writers regarded the disease as a variety or modification of Typhus. From pernicious intermittents and remittents, it differs still more widely. And certainly the absence of all soreness of the throat, swelling of the glands of the neck, exanthematous rash, and desquamation of the cuticle, should prevent it from being confounded with scarlatina.

*Post Mortem Appearances.*—The effects of this disease, or the appearances after death, vary much, according to the duration of the disease. In those cases terminating fatally, within the first twenty-four hours, little or no structural change is visible in any part of the body. The blood is dark and its coagulability impaired; the veinous capillaries generally congested or full, especially in the base and posterior parts of the brain and its membranes; and sometimes ecchymosed spots in the interior of the larger bloodvessels. If death has taken place at a later period of the disease, the membranes of the base and posterior parts of the brain are generally intensely red, with effusions both plastic and serous; the cerebral substance also injected and often softened; the muscular structures darker color and softer than natural; the lungs often passively engorged; and the kidneys often exhibiting appearances of fatty degeneration or the accumulation of fat globules in their cortical substance. The urine has often been found albuminous, both before and after death.

*Pathology.*—Such is a very brief glance at the symptoms, progress, and results of one of the most fatal diseases with which the physician has to contend. But what is its nature?

Where is its primary seat? And what are the correct indications for treatment? These are the all-important, all-absorbing questions with the practitioner at the bedside of his patient. Some regard it as a simple active inflammation of the medulla oblongata and base of the brain with their investing membranes. And hence the name "cerebro-spinal meningitis." That such local inflammation does occur in the progress of the disease, when it does not prove fatal in the first few hours, is clearly proved both by the symptoms and *post mortem* appearances. But is this the primary and essential part of the disease? Or is it merely an accompaniment, like the sore throat in scarlatina, the pustules in variola, or the affection of Peyer's glands in typhoid fever?

If we concede that the cerebro-spinal inflammation is primary and constitutes *the* disease, is that inflammation sthenic or asthenic? Are the properties of the structures involved, exalted, or impaired?

But a large proportion of those who have expressed opinions in relation to the disease, during the last few years, claim that the local inflammation is secondary; that the primary disease consists in a poisoned condition of the blood. "Blood-poisoning" has become a very fashionable term in modern pathology, and bids fair to supersede the magic word "*bilious*," which has so long served to explain all the ills of life.

But on the supposition that the disease, or its active phenomena, depends on a toxic or poisoned condition of the blood, what is the nature and whence the origin of the poison? There are two modes by which the blood may become impure or poisoned.

First, by the introduction of poisonous agents from without; as by the inhalation of an atmosphere impregnated with deleterious agents; by absorption of poisonous material from unhealthy, suppurating, and gangrenous surfaces; and by direct inoculation with poisonous viruses.

Second, by a failure of one or more of the excretory organs to eliminate from the blood the effete material as fast as it is derived from the metamorphosis of tissues. Thus a failure in the healthy action of the skin, lungs, liver, and kidneys causes a corresponding failure in the secretion and elimination of perspiratory matter, of carbonic acid gas, of cholesterine and coloring matter of bile, and of urea, &c., and consequently an accumulation of these substances in the blood. There are two ways in which the deleterious agents introduced into the blood by either of the above methods, may produce their morbid effects. First, by acting directly on one or more organs or structures by contact, the blood being only a menstruum for holding the poison in solution and conveying it to the different parts of the system. Secondly, by acting primarily on the constituents of the blood itself, in such a manner as to impair their properties and render them incapable of performing their functions; and in some instances, at least, the action on the constituents of the blood is such, as to rapidly reproduce or multiply the original poison, as in the contagious fevers. When a deleterious agent acts in accordance with the first of these methods, the result is not properly a *blood disease;* but when its action is in accordance with the second method, it produces a true "blood poisoning," or "blood disease." It matters not whether the poison enters into actual combination with one or more of the ingredients of the blood, or whether it only induces morbid changes by its presence, analogous to well known catalytic action out of the living system, it, in either case, produces a primary blood disease. If cerebro-spinal meningitis or spotted fever depends on the action of a distinct or specific poison, does it originate exteriorly and gain access to the blood by inhalation and absorption; or does it originate from some combination of effete material retained within, from defect in the excretory functions?

When the poison is actually in the blood, does it produce the active phenomena of disease by acting directly on one or more of the organized structures, or primarily on the blood itself? These questions are both interesting and practically important. But observations hitherto made, concerning the circumstances under which the disease occurs, are so defective that a perfectly satisfactory answer cannot be given in the present state of our knowledge. We shall never be able to trace, satisfactorily, the causes of epidemics, until accurate registries have been kept in considerable numbers, containing an exhibit of the thermometrical, hygrometrical, and electrical conditions of the atmosphere at the time such diseases make their appearance and during their prevalence. We need also a record of the previous physical condition and habits, and the condition of the lodging rooms, of each individual attacked, in any given epidemic. With an exact knowledge of all the important atmospheric conditions, and of the personal hygiene of the individuals attacked, it is highly probable that the efficient cause of the disease could be made apparent.

From the fact that the disease has prevailed more in rural districts than in cities; more frequently after extreme cold weather than at other periods; more with the young than the aged; while the topography of the several districts in which it has occurred is widely diverse; it is difficult to conceive of the production and diffusion of any one specific atmospheric poison under circumstances so variable.

And yet the suddenness with which the disease attacks its victim; the rapidity of its progress; and the uniformity with which it leaves traces of morbid action in certain structures; certainly indicate the action of some subtile and energetic poison. Whether it is derived from without or generated within, its primary action seems to be directly on the *vital affinity* of the tissues, so far suspending it as to impair all the organic changes, and thereby retard the movements of the blood in the whole capillary system of bloodvessels, indicated by rapid suspension of one function after another and the early appearance of purple or ecchymosed spots. But while the cause thus evidently influences generally that property of the living structures which we call *vital affinity*, it plainly manifests a special tendency to act on the serous surfaces, more especially those investing the cerebro-spinal portion of the nervous tissues, but sometimes also the pericardium, pleura, and endangium or interior lining of the vascular system. And hence it is, that, in all cases except those cases which terminate fatally in a few hours, we find evidences of severe disease in one or more of these membranous structures. In the sudden general derangement of the system, followed so speedily by the tendency to asthenic inflammation in the cerebro-spinal meninges, or other internal membranous structures; as well as the frequent prevalence of the disease coincidently with that of erysipelas in the same neighborhood, there is suggested a close analogy between the two diseases.

*Treatment.*—But whatever theories we may adopt in relation to the disease in question, it is evident, from both symptoms and *post mortem* phenomena, that there are two principal sources of danger to the patients. First, from direct suspension of organic actions and death before reaction or local inflammation can be developed. Second, from the supervention of inflammation, chiefly, but not exclusively, in the cerebro-spinal centres. Such inflammation being of an asthenic character, its danger is greatly increased by the general derangement both of the blood and the properties of the solids. Whether we regard the severe and fatal epidemic which has prevailed in many localities during the past year, as a primary local inflammation, as a general disease of zymotic origin (that is, from occult atmospheric poison absorbed into the blood), complicated with local cerebro-spinal inflammation; or as a local disease of the excretory organs, allowing the blood to become poisoned by retention of urea or any other effete material, the real indications to be fulfilled by the practitioner at the bedside are, first, to maintain the vital properties, and consequently the atomic or organic changes throughout the various structures of the body; second, to restore general secretion, thereby eliminating whatever poison may exist in the blood, or to neutralize it without elimination; third, to counteract the known tendency to the development of dangerous local inflammation; and fourth, to combat such local inflammation after it is developed. The relative importance of these indications differs according to the severity of each individual case. In those severe and highly malignant attacks, in which the life of the patient is endangered by the direct and immediate suspension of the functions, not only of the cerebro-spinal centres but also of capillary and secretory actions generally, the two first indications are of paramount importance. But in all those cases in which the attack is less severe and febrile reaction becomes readily established, the two last indications assume the highest importance. While we feel no doubt about the general correctness of the foregoing indications for treatment, we freely acknowledge a feeling of embarrassment in recommending the individual remedies most efficient for fulfilling them. This embarrassment is increased by the well-known fact that the success of almost every remedy depends, not simply on its abstract qualities, but very much on the time and mode of its use.

The practitioner standing at the bedside of a patient, just prostrated by a chill or condition of extreme organic depression, and realizing that every hour is carrying him nearer to the point of fatal suspension of all the functions, what can he do to arouse the nervous, capillary, and secretory susceptibilities, so promptly as to restore the functions of these various structures, and thereby carry the patient safely beyond the first and most dreaded period of danger? Perhaps no one agent judiciously managed exerts more speedy and direct influence over the elementary properties of living tissues, whether nervous, vascular, or secretory, than free caloric and electricity. It has long been known, especially among the more eminent practitioners in the States bordering on the lower Mississippi and Gulf of Mexico, that one of the most efficient means for arousing the susceptibilities of the system, so as to establish reaction in pernicious intermittents, consists in thorough dashing of cold water over the whole surface, with alternate wrapping in warm blankets. The same process was found more successful than any other, for the same purpose, by Dr. ANDERSON, of Alabama, in one of the most malignant and fatal epidemics of scarlatina that ever visited that section of the country. We would suggest a modified form of the application of this remedy in the first stage of cerebro-spinal meningitis, or spotted fever. We would apply cold, by bags of pounded ice, if possible, to the occiput, cervical and upper part of the dorsal spine, and at the same time cloths wet in water as warm as can be borne by the hand, constantly to the whole epigastric and abdominal regions, and also to the lower extremities. At the same time, we would administer internally, not diffusable stimulants or inebriants, nearly all of which, either directly or indirectly, retard the play of vital affinities, and consequently of organic or atomic changes in the tissues, but true organic stimulants.

Among the most active and reliable of these, are oxygen, phosphorus, cantharides, guaiac, turpentine, arsenic, and the chlorine and mineral acid salts.

The first, from its gaseous form and consequent inconvenient portability, can never be made extensively available in practice. The second, phosphorus, from its strong affinity for oxygen and consequent spontaneous inflammability, must also be excluded, to a great extent, from general practice; while its compounds, whether phosphates or phosphites, are comparatively inactive. No such objections, however, can be made to the remaining articles enumerated. A well-prepared tincture of cantharides constitutes one of the most speedily acting and efficient organic stimulants that we possess; and, in the stage of depression at the outset of the disease, we would give it in doses of from 20 to 30 minims; first, every hour; and subsequently, every two, three, or four hours, according to the effects.

If erysipelas was prevailing to any extent in the same locality, coincidently with the disease under consideration, we would add to each dose of the tincture of cantharides an equal quantity of the muriated tinct. of iron. In addition to either one or both of these remedies, we would also give moderately full doses of a solution of either chlorate of potassa or sulphite of soda, combined with tinct. of belladonna. A solution of about fifteen grains of either of these salts, with the same number of minims of the tincture of belladonna, may be given between each of the doses of cantharides.

The objects designed to be accomplished by this combination are three, namely, to increase the capacity of the blood for holding in solution oxygen gas, and thereby induce a greater absorption of that powerful organic stimulant from the inspired air in the pulmonary air cells; to lessen the tendency to septic or necremic changes in the blood itself; and to induce contraction of the capillary vessels of the cerebro-spinal nervous centres, so as to relieve them of that passive congestion which we believe constitutes the first step in the development of the severe inflammation which so generally supervenes in that locality. That the salts here named have the power to increase the capacity of the blood for absorbing and holding in solution oxygen, has been clearly demonstrated both by experiment and clinical observation. Several years since, M. Bernard, by a well-devised series of experiments, fully established the fact, that solutions of nearly all the saline compounds, when present in the blood out of the body, increased in a notable degree the capacity of that fluid for the absorption of oxygen.

Since those experiments, several cases of cyanosis have been reported, in which a free exhibition of chlorate of potassa in solution, promptly changed the venous color of the blood to an arterial hue, so long as the salt remained in the blood. We have also several times obtained the same results in two cases of cyanosis from defective closure of the foramen ovale of the heart. The result is not obtained, as some have supposed, by the liberation of a portion of the oxygen of the salt, for the latter is soon eliminated through the kidneys, unchanged in its composition. That this power to increase the capacity of the blood for absorbing oxygen from the air in the lungs, is a very important one, not only in the depressed stage of the disease under consideration, but in certain stages in many other affections, few reflecting pathologists will deny.

That these salts, and especially the sulphites, are capable of retarding or arresting a septic or necremic change in the blood, we have sufficiently shown in another part of this report. The last effect proposed to be accomplished by remedies, namely, contraction of the capillaries of the cerebro-spinal centres, depends on the belladonna.

We presume all the members of the Society are familiar with the experiments of Brown-Séquard for determining the action of certain substances on the cerebral circulation and functions, by which he deduced the fact that belladonna, stramonium, and ergot, were each capable, when taken internally, of inducing contraction of the cerebro-spinal capillaries, and thereby diminishing the amount of blood in them, together with his application of the knowledge of this fact to the treatment of certain forms of paralysis. If the results of his observations are correct, and our clinical experience has thus far confirmed them, no remedy could be selected calculated to so directly counteract the development of cerebro-spinal inflammation or congestion, as the belladonna. Such are the means and the reasons for their use, on which we would rely for the treatment of the early stage of this most formidable disease. If they meet this stage successfully, the disease is arrested and convalescence ensues, without the interposition of other remedial agents.

But if these remedies were only partially successful, or we were not called until the first stage had passed, and the cerebro-spinal inflammation was more fully developed with general febrile reaction, we should omit the cantharides but continue all the other remedies and applications recommended in the first stage, with the addition of thorough dry cupping over the cervical and dorsal portions of the spine, and the cautious administration of alteratives and diuretics, being cautious to avoid active purgation or any other strongly depressing influence. Such are our views of the treatment of this formidable malady, based, we admit, upon rational, or, if you choose, theoretical, deductions. And yet they are not wholly without the practical test of experience. For we have been called to treat five strongly-marked cases of this disease during the past year.

We would not leave the impression, that the plan of treatment here recommended, or any other, will cure every case of cerebro-spinal meningitis or spotted fever. For we are well aware that in all severe epidemics, whether of typhus, yellow fever, scarlatina, variola, erysipelas, spotted fever, or cholera, cases occur in which, from the first hour of the attack, the elementary properties of the tissues, on which all organic changes depend, are so profoundly depressed, or, more properly, so nearly suspended, that no remedial agents will make any impression whatever. But do or give whatever you please, the various functions become steadily more and more feebly performed, until in a very few hours life becomes extinct—leaving but little visible traces of morbid action in either the solids or fluids of the body.

